# An Aqueous Facile Synthesis of 2,3-Dihydroquinazolin-4(1H)-One Derivatives by Reverse Zinc Oxide Micelles as Nanoreactor

**DOI:** 10.3389/fchem.2020.00239

**Published:** 2020-04-24

**Authors:** Jie Mou, Ninghai Chen, Yu Zhao, Hao Qi, Sihan Meng, Rui Xiang, Dongsheng Pei

**Affiliations:** ^1^Jiangsu Key Laboratory of New drug and Clinical Pharmacy, Xuzhou Medical University, Xuzhou, China; ^2^School of Pharmacy, Xuzhou Medical University, Xuzhou, China; ^3^Department of Pathology, Xuzhou Medical University, Xuzhou, China

**Keywords:** green chemistry technology, synthetic method, 2,3-dihydroquinazolin-4(1H)-one, aqueous media, reverse zinc oxide micelle, nanoreactor

## Abstract

A green synthetic protocol has been developed for the efficient preparation of 2,3-dihydroquinazolin−4(1H)-one derivatives with excellent yield in aqueous media. Reverse zinc oxide micelles catalyzed the reactions efficiently and selectively as the hallow nanoreactor. Moreover, the catalyst was reusable without significant loss of catalytic efficiency. The notable advantages of the procedure are high yields and mild reaction conditions, simple operation, nonchromatographic purification, environmentally friendly and good versatile substrates.

## Introduction

N-heterocycles are important integral pharmacophoric units ubiquitously used in a variety of biologically active natural products, agrochemicals, pharmaceutical and synthetic drugs (Khan et al., 2016). 2,3-Dihydroquinazolin-4(1H)-one plays an important role in the aromatic nitrogen-containing heterocycles because of their abundant pharmacological and biological activities (Kshirsagar, 2015; Zawawi et al., 2015; Dohle et al., 2018). Compounds containing this motif have shown significant biological activities which include anticancer, anticonvulsant, antidefibrillatory, analgesic, diuretic, antihistamine, antihypertensive, and many other activities (Patil et al., 2015; Zawawi et al., 2015). These heterocycles participate in physiological processes as markers or messenger molecules and a large number of pharmaceuticals based on these heterocycles have been reported in the past few decades (Saeedi et al., 2018). For example, N^1^-substituted-2,3-dihydroquinazolin-4(1H)-one (I) is a cholinesterase inhibitor (Sultana et al., 2017), 1,7-disubstituted-2,3-dihydroquinazolin-4(1H)-one (II) is a selective PKC inhibitor (Katoh et al., 2016), bis(2,3-dihydroquinazolin-4(1H)-one (III) showed potent radical scavenging activities (Sivaguru et al., 2017), 2,7-disubstituted-2,3-dihydroquinazolin-4(1H)-one (IV) is a TRPM2 inhibitor (Zhang et al., 2018), 2-disubstituted-2,3-dihydroquinazolin-4(1H)-one (V) showed antitubulin activity (Singh and Raghav, 2015), and 2,2-disubstituted-2,3-dihydroquinazolin-4(1H)-one (VI) is considered as a potential lead compound as dual AChE/BChE inhibitor (Sarfraz et al., 2017) Figure 1.

**Figure 1:**
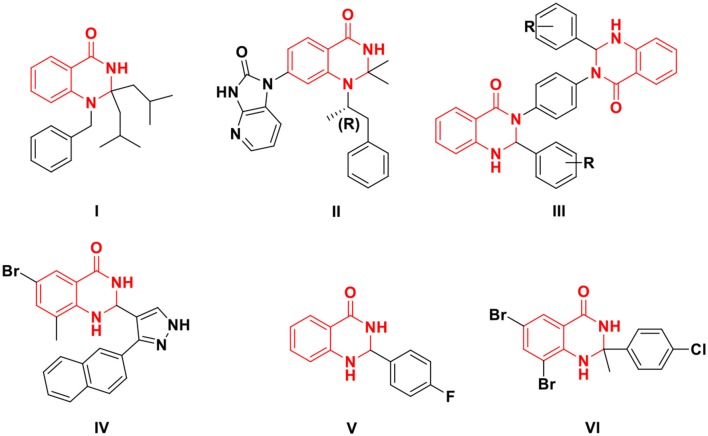
Important lead compounds with 2,3-dihydroquinazolin-4(1H)-one skeleton.

Owing to wide range of applications, several approaches for the preparation of 2,3-dihydroquinazolin-4(1H)-one have been reported (Pospisilova et al., 2018; Pathare et al., 2019). The preferable route is to directly catalyze the condensation of anthranilamide with aldehydes for target products (Xing et al., 2017). However, most of these approaches are associated with numerous limitations including complicated reactions, vast excess of oxidant, non-renewable solvents, harsh reaction conditions (such as temperatures of up to 100°C), long reaction time and low reaction yields (Bie et al., 2016; Parua et al., 2017; Sun et al., 2018; Wang et al., 2018). In recent years, the focus has been on the development of economical and convenient methods for the synthesis of dihydroquinazolin-4(1H)-one derivatives via a tolerable approach (Tamaddon and Kazemivarnamkhasti, 2016; Liu et al., 2018). Additionally, an efficient and highly selective catalyst is definitely indispensable. Wu et al. (2013) developed an efficient method for the preparation of dihydroquinazolinones analogs by employing ZnCl_2_ as a catalyst in EtOH. However, the percentage of product yielded is lower than 50%. To our delight, the group IIB transition metal Lewis acids are promising catalyzers for this transformation. Nano-sized metal oxides usually undergo agglomeration when they are dispersed in solutions due to their large specific surface area and high surface activity (Punnoose et al., 2014; Curran et al., 2017; Liu et al., 2018), which has a major impact on their reactivity. And the porous nanomaterial was found to be an efficient, selective and waste-free green approach (Rostamnia and Xin, 2014). Catalyst-containing hollow nanocapsules have attracted particular interest because they provide the storage spaces or reaction chambers for a diffusional product/substrate exchange between the inner cavity and the bulk solution to take place efficiently (Sanles-Sobrido et al., 2012). Consequently, we designed the reverse zinc oxide nanomicelles as functional catalyst nanoreactors to achieve the dual purpose of both stabilizing the dispersal and providing nano-size control.

**Scheme 1:**
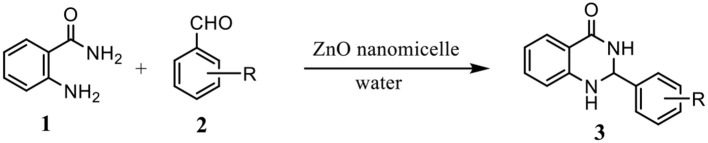
Synthesis of 2,3-dihydroquinazolin−4(1H)-one derivatives.

Our previous studies have shown that the Betti reaction could be promoted by reverse zinc oxide micelles in aqueous efficiently and selectively (Mou et al., 2017). As interest continues in the potential use of such a catalyst, we report herein a simple, one-pot synthesis of 2-substituted 2,3-dihydroquinazolin-4(1H)-one from substituted aldehydes and anthranilamide in the presence of reverse zinc oxide micelles as a catalytic system (Scheme 1).

## Materials and Methods

All solvents and reagents were commercial and used without further purification. All terminal products were confirmed by mp, FT-IR spectroscopy, ^1^H NMR and HRMS. Melting points were measured in open capillary tubes and were uncorrected. FT-IR spectra were obtained on an infrared spectrophotometer (Jasco FT-IR 4100 Series, Perkin-Elmer, USA) using KBr disks. ^1^H NMR spectra were recorded on a JNM-ECZ400s/L spectrometer (400 MHz, Joel, Japan) using CDCl_3_ or DMSO-*d*_6_ as the deuterated solvent. The chemical shifts have been reported in (ppm) downfield relative to (Me)_4_Si. High-resolution-mass spectra (HRMS) analyses were recorded at room temperature on a micrOTOF-Q instrument (Bruker, USA).

### Preparation and Characterization of Catalyst

**Scheme 2:**
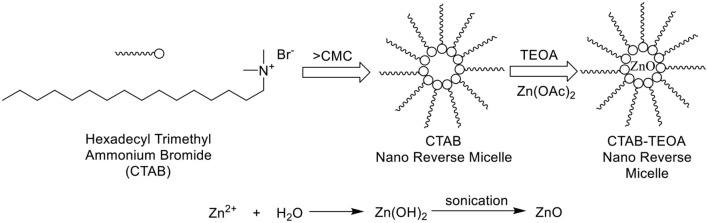
Preparation of reverse ZnO nanomicelles.

The reverse zinc oxide nanomicelles was synthesized following the reported procedure (Mou et al., 2017) (Scheme 2). Reverse micelles were formed by the self-assembly of the surfactant in cyclohexane. The particle size and distribution of reverse ZnO nanomicelles were recorded on NICOMP 380ZLS particle size analyzer (PSS, USA). The morphology was illustrated using transmission electron microscopy (TEM, Joel, Japan). The micelles were dispersed via ultrasonication in water for 1 min before deposition on the TEM grid. X-ray diffractometers (XRD) were conducted on a Rigaku D/max-III type instrument (Denki, Japan) using Cu Kα (1.54 Å) radiation with a scanning speed of 4°/min from 5° to 90°.

### General Procedure for Preparation of 2,3-dihydroquinazolin-4(1H)-ones 3a−3l

To a solution of the catalyst in H_2_O (10 mol%, 5 mL), anthranilamide (0.1 mmol) and substituted aromatic aldehydes (0.1 mmol) were added. The resulting mixture was stirred under 70°C for a period of time. The reaction was monitored by thin layer chromatography (TLC). After completion of the reaction, the precipitate was filtered and recrystallized from ethanol (95%) to obtain the pure target product. The catalyst remaining in the water filter liquor could be used directly as a catalyst media for subsequent runs.

## Results

### Characterization of Reverse ZnO Nanomicelles

**Figure 2:**
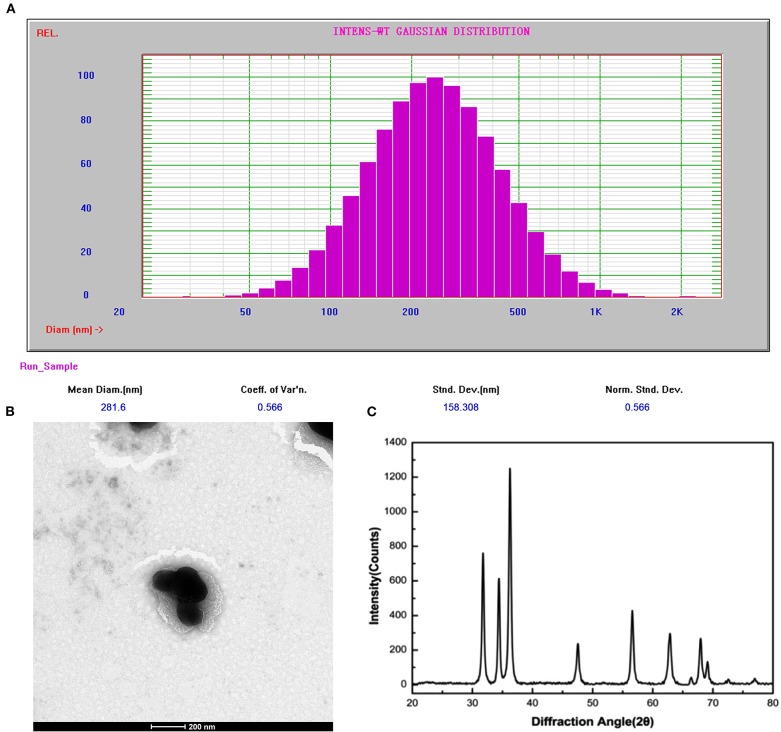
**(A)** Particle size and distribution of reverse ZnO nanomicelles by PSS. **(B)** TEM image of reverse ZnO nanomicelles. **(C)** XRD spectrum of ZnO nanoparticles.

The distribution of sphere diameters and TEM image are shown in Figures 2A,B, respectively. The image revealed that the average diameter was 280 nm and the size distribution was about 60%. The XRD pattern of ZnO nanoparticles calcined at 400°C is shown in Figure 2C. The diffraction peaks appeared at 2θ value similarly to the hexagonal structure of zincite phase reported in JCPDS File Card No. 05-0664.

### Synthesis of 2,3-dihydroquinazolin-4(1H)-one Derivatives

#### 2-(4-nitrophenyl)-2,3-dihydroquinazolin-4(1H)-one (3a)

Yellow solid; mp: 158–161°C, Lit 165–168°C (Ramesh et al., 2017); IR(KBr, ν, cm^−1^): 3340, 1670, 1586, 1520, 1445, 1344, 1296, 1186; ^1^H NMR (400 MHz, CDCl_3_) δ 8.55 (s, 2H), 8.40–8.35 (m, 2H), 8.32 (dd, *J* = 7.9, 2.0 Hz, 1H), 8.05 (dd, *J* = 9.2, 2.5 Hz, 2H), 7.58–7.53 (m, 1H), 7.46–7.41 (m, 1H), 7.08 (dd, *J* = 7.9, 2.0 Hz, 1H), 5.97 (s, 1H). HRMS (ESI) calcd for [C_14_H_11_N_3_O_3_-H^+^]: 268.0717, found: 268.0725.

#### 2-(3-nitrophenyl)-2,3-dihydroquinazolin-4(1H)-one (3b)

Yellow solid; mp: 166–169°C, Lit 163–165°C (Shaabani et al., 2008); IR(KBr, ν, cm^−1^): 3337, 1674, 1588, 1526, 1447, 1353, 1271, 1188; ^1^H NMR (400 MHz, CDCl_3_) δ 8.70 (t, *J* = 2.0 Hz, 1H), 8.54 (s, 1H), 8.45–8.39 (m, 2H), 8.32 (dd, *J* = 8.0, 1.6 Hz, 1H), 8.21 (d, *J* = 7.8 Hz, 1H), 7.73 (t, *J* = 8.0 Hz, 1H), 7.58–7.53 (m, 1H), 7.45–7.41 (m, 1H), 7.07 (dd, *J* = 8.0, 1.6 Hz, 1H), 5.92 (s, 1H). HRMS (ESI) calcd for [C_14_H_11_N_3_O_3_-H^+^]: 268.0717, found: 268.0718.

#### 2-(2-chlorophenyl)-2,3-dihydroquinazolin-4(1H)-one (3c)

White solid; mp: 208–210°C, Lit 207–209°C (Ghafuri et al., 2019); IR(KBr, ν, cm^−1^): 3362, 1647, 1614, 1503, 1330, 1253, 1055; ^1^H NMR (400 MHz, CDCl_3_) δ 7.91 (dd, *J* = 7.8, 1.6 Hz, 1H), 7.79–7.67 (m, 1H), 7.42–7.38 (m, 1H), 7.36–7.26 (m, 3H), 6.89–6.85 (m, 1H), 6.66 (dd, *J* = 7.8, 1.6 Hz, 1H), 6.34 (t, *J* = 1.8 Hz, 1H), 6.05 (s, 1H), 4.63 (s, 1H). HRMS (ESI) calcd for [C_14_H_11_ClN_2_O-H^+^]: 257.0476, found: 257.0457.

#### 2-(3-bromophenyl)-2,3-dihydroquinazolin-4(1H)-one (3d)

White solid; mp: 183–185°C, Lit 184–185°C (Khoshnavazi et al., 2016); IR(KBr, ν, cm^−1^): 3288, 1647, 1614, 1514, 1299, 1195, 1070; ^1^H NMR (400 MHz, CDCl_3_) δ7.93 (dd, *J* = 8.0, 1.5 Hz, 1H), 7.77 (s, 1H), 7.59–7.56 (m, 1H), 7.51 (d, *J* = 7.8 Hz, 1H), 7.37–7.28 (m, 2H), 6.91 (t, *J* = 7.5 Hz, 1H), 6.67 (d, *J* = 8.0 Hz, 1H), 5.87 (s, 1H), 5.80 (s, 1H), 4.38 (s, 1H). HRMS (ESI) calcd for [C_14_H_11_BrN_2_O-H^+^]: 300.9971, found: 300.9987.

#### 2-(4-hydroxyphenyl)-2,3-dihydroquinazolin-4(1H)-one (3e)

White solid; mp: 182.6–184.9°C, Lit 192–194°C (Dindulkar et al., 2014); IR(KBr, ν, cm^−1^): 3288, 1647, 1614, 1514, 1299, 1195, 1070; ^1^H NMR (400 MHz, DMSO-*d*_6_) δ 9.45 (s, 1H), 8.04 (s, 1H), 7.58–7.55 (m, 1H), 7.29–7.16 (m, 3H), 6.89 (s, 1H), 6.76–6.60 (m, 4H), 5.61 (s, 1H). HRMS (ESI) calcd for [M-H^+^]: 239.0815, found: 239.0796. HRMS (ESI) calcd for [C_14_H_12_N_2_O_2_-H^+^]: 240.3562, found: 240.0795.

#### N-(4-(4-oxo-1,2,3,4-tetrahydroquinazolin-2-yl)phenyl)acetamide(3f)

White solid; mp: >220°C, Lit 241–242°C (Rostamizadeh et al., 2010); IR(KBr, ν, cm^−1^): 3330, 1680, 1649, 1610, 1509, 1312; ^1^H NMR (400 MHz, DMSO-*d*_6_) δ 9.94 (s, 1H), 8.20 (s, 1H), 7.61–7.50 (m, 3H), 7.37 (d, *J* = 8.5 Hz, 2H), 7.25–7.16 (m, 1H), 6.97 (s, 1H), 6.69 (t, *J* = 7.1 Hz, 1H), 6.64 (t, *J* = 7.4 Hz, 1H), 5.66 (s, 1H), 2.00 (s, 3H). HRMS (ESI) calcd for [C_16_H_15_N_3_O_2_-H^+^]: 280.1081, found: 280.1089.

#### 4-(4-oxo-1,2,3,4-tetrahydroquinazolin-2-yl)phenyl acetate(3g)

White solid; mp: 193–194°C; IR(KBr, ν, cm^−1^): 3300, 1768, 1654, 1613, 1507, 1390, 1217; ^1^H NMR (400 MHz, CDCl_3_) δ 7.92 (dd, *J*_1_ = 7.8, 1.5 Hz, 1H), 7.64–7.52 (m, 2H), 7.35–7.30 (m, 1H), 7.20–7.10 (m, 2H), 6.91–6.87 (m, 1H), 6.65 (dd, *J*_1_ = 7.8 Hz, *J*_2_ = 1.5 Hz, 1H), 5.89 (s, 1H), 5.80 (s, 1H), 4.4(s, 1H), 2.31 (s, 3H). HRMS (ESI) calcd for [C_16_H_14_N_2_O_3_-H^+^]: 281.0921, found: 281.2468.

#### 2-(2-(trifluoromethyl)phenyl)-2,3-dihydroquinazolin-4(1H)-one(3h)

White solid; mp: 188–190°C, Lit 187–188°C (Dutta et al., 2019); IR(KBr, ν, cm^−1^): 3356, 1671, 1610, 1507, 1484, 1230, 1210; ^1^H NMR (400 MHz, CDCl_3_) δ 7.93 (dd, *J* = 7.8, 1.5 Hz, 1H), 7.54–7.45 (m, 2H), 7.34–7.30 (m, 1H), 6.99–6.82 (m, 3H), 6.65 (d, *J* = 7.9 Hz, 1H), 5.84 (s, 1H), 5.71 (s, 1H), 4.35 (s, 1H). HRMS (ESI) calcd for [C_13_H_11_N_3_O-H^+^]: 224.0818, found: 224.0831.

#### 2-(pyridin-2-yl)-2,3-dihydroquinazolin-4(1H)-one(3i)

White solid; mp: 172–175°C; IR(KBr, ν, cm^−1^): 3319, 3076, 1656, 1613, 1510, 1312, 1279, 1132, 1122, 1161, 772; ^1^H NMR (400 MHz, CDCl_3_) δ 8.15 (d, *J* = 7.8 Hz, 1H), 7.89 (dd, *J* = 7.8, 1.3 Hz, 1H), 7.73–7.59 (m, 2H), 7.52 (t, *J* = 7.7 Hz, 1H), 7.36–7.28 (m, 1H), 6.90–6.86 (m, 1H), 6.65 (dd, *J* = 7.8, 1.3 Hz, 1H), 6.35 (s, 1H), 5.92 (s, 1H), 4.44(s, 1H). HRMS (ESI), calcd for [C_15_H_11_F_3_N_2_O-H^+^]: 291.0740, found: 291.0747.

#### 2-(4-methoxyphenyl)-2,3-dihydroquinazolin-4(1H)-one (3j)

Brown solid; mp: 184–185°C, Lit 182–184°C (Katla et al., 2017); IR(KBr, ν, cm^−1^): 3292, 2851, 1665, 1613, 1514, 1256, 1066; 1H NMR (400 MHz, CDCl_3_) δ 8.61–8.59 (m, 1H), 7.91 (dd, *J* = 7.8, 1.6 Hz, 1H), 7.78–7.73 (m, 1H), 7.59 (d, *J* = 7.9 Hz, 1H), 7.32–7.29 (m, 2H), 6.89–6.85 (m, 1H), 6.71 (d, *J* = 7.2 Hz, 1H), 6.52 (s, 1H), 5.91 (t, *J* = 2.3 Hz, 1H), 5.00 (s, 1H), 3.71 (s, 3H). HRMS (ESI) calcd for [C_15_H_14_N_2_O_2_-H^+^]: 253.0972, found: 253.0973.

#### 2-(2,4-dimethoxyphenyl)-2,3-dihydroquinazolin-4(1H)-one(3k)

White solid; mp: 178–181°C, Lit 182–183°C (Hour et al., 2000); IR(KBr, ν, cm^−1^): 3425, 3298, 2837, 1653, 1613, 1509, 1256, 1033; ^1^H NMR (400 MHz, CDCl_3_) δ 7.90 (dd, *J* = 7.8, 1.5 Hz, 1H), 7.47 (d, *J* = 8.1 Hz, 1H), 7.32–7.25 (m, 1H), 6.89–6.77 (m, 1H), 6.65–6.57 (m, 1H), 6.51–6.40 (m, 2H), 6.17 (t, *J* = 1.6 Hz, 1H), 5.87 (s, 1H), 4.54 (s, 1H), 3.84 (s, 3H), 3.80 (s, 3H). HRMS (ESI), calcd for [C_16_H_16_N_2_O_3_-H^+^]: 283.1077, found: 283.2685.

#### 2-(4-hydroxy-3,5-dimethoxyphenyl)-2,3-dihydroquinazolin-4(1H)-one(3l)

White solid; mp: >220°C; IR(KBr, ν, cm^−1^): 3365, 3334, 2835, 1663, 1612, 1511, 1243, 1204; ^1^H NMR (400 MHz, CDCl_3_) δ (ppm): δ 7.93 (d, *J* = 7.7 Hz, 1H), 7.35–7.32 (m, 1H), 6.90 (t, *J* = 7.5 Hz, 1H), 6.82 (s, 2H), 6.68 (d, *J* = 8.0 Hz), 5.80 (s, 1H), 5.76 (s, 1H), 5.67 (s, 1H), 4.38 (s, 1H), 3.91 (s, 6H). HRMS (ESI) calcd for [C_13_H_18_N_2_O_3_-H^+^]: 299.1026, found: 299.1018.

## Discussion

### Optimization Studies

The catalytic activity of reverse ZnO nanomicelles in the preparation of 2,3-dihydroquinazolin-4(1H)-ones was investigated after the catalyst characterization. In this respect, the cyclocondensation of anthranilamide and 4-nitrobenzaldehyde was selected as a model reaction to optimize the reaction condition by varying solvent, catalyst amount and reaction temperature. The results were exhibited in Table 1. It was found that the catalyst amount plays a vital role in the product yield and conversion time (Table 1, entries 1–5). The reaction was initially employed in water at room temperature for 1 h with 3% catalyst dosage, the target product 3a was obtained with 71% yield (entry 1). The yield was increased from 70 to 99% with the increasing amount of catalyst, the highest yield reach 99% when 10% reverse ZnO nanomicelles were applied. The reaction time was greatly shortened by increasing the amount of catalyst, but the yield did not increase significantly more than 10 mol% ZnO nanomicelles added. It is indicated that the desired product 3a was obtained by catalytic amounts of reverse ZnO nanomicelles. Notably, the terminal product was isolated by filtration and recrystallazation with excellent yield without any column purification.

**Table 1:** Optimization of reaction condition.

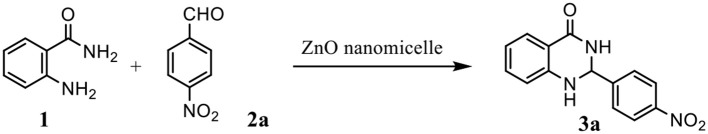					
**Entry**	**Catalyst (mol%)**	**Solvent^a^**	**Temp. (°C)**	**Time (min)**	**Yield (%)^b^**
1	3	Water	25	60	71
2	5	Water	25	45	82
3	10	Water	25	30	71
4	15	Water	25	10	95
5	20	Water	25	8	95
6	10	Water	60	5	90
7	10	Water	70	5	89
8	10	Water	90	5	95
9	10	CH_2_Cl_2_	25	120	28
10	10	THF	60	120	21
11	10	DMF	80	120	14
12	10	EtOH	60	120	16

a *Reactions were conducted in solvent 5.0 mL on a 1 mmol scale at the special temperature using 10 mol% reverse ZnO nanomicelles as catalyst, 12 h*.

b *Isolated yields*.

Subsequently, the feasibility of the strategy to various solvents was investigated. The organic solvents such as dichloromethane (CH_2_Cl_2_), tetrahydrofuran (THF), dimethyl formamide (DMF), ethanol (EtOH) were examined. Surprisingly, although raw materials dissolved perfectly in these organic solvents, none of them affords higher yield of the target product than water (entries 9–12). An almost quantitative yield was achieved within 2 h when the reaction was conducted in organic solvents. Even the best of them, dichloromethane, only afforded 28% yield (Table 1, entry 9), which is about one third of the yield under the same condition in water (Table 1, Entry 1). From these results, it was evident that water is most suitable for the synthesis of 3a in the presence of reverse zinc oxide micelle. This phenomenon may be a result of the unusual feature of the reverse zinc oxide micelle and the reaction mechanism which will be further discussed.

### Stability of the Catalytic System

**Figure 3:**
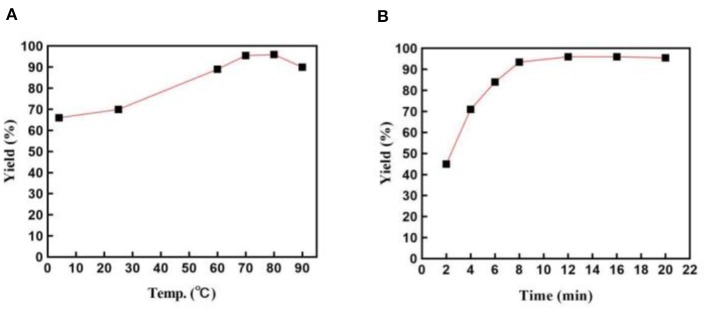
**(A)** Effects of the reaction temperature on the preparation of **3a**. Reactions were employed on a 1 mmol scale; reverse ZnO nanomicelles 10 mol%; **(B)** Influence of the reaction time on the yield of **3a**. Reactions were conducted on a 1 mmol scale; reverse ZnO nanomicelles 10 mol%, 70°C.

We then investigated the relationship between temperature and the product yield (Figure 3A). Notably, this system had extraordinary reaction activity and an ability to give a good yield at room temperature or low temperature (4°C). The highest yield was obtained at 80°C. However, taking energy consumption into consideration, we thought 70°C was the most suitable temperature for the reaction, as it cost less, despite producing slightly less yield than 80°C did. When the temperature elevated to 90°C, the yield declined possibly due to the micelles' loss of surface area caused by dissociation. Additionally, we explored the best reaction time (Figure 3B). As is shown in Figure 3B, the reaction proceeded quickly in 8 min, reaching a fairly high yield. No obvious increase of the yield was detected after the reaction was conducted for 8 min.

**Figure 4:**
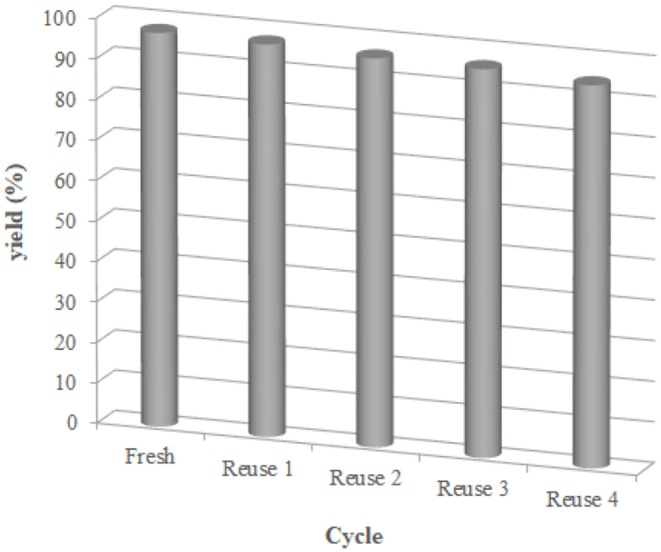
Reusability of reverse ZnO micelles in the preparation of **3e**. Reaction conditions: 1 (1 mmol), **2e** (1 mmol), catalyst (reverse ZnO nanomicelles, 10 mol%), solvent (water, 5 mL), 70°C, 12 min. Isolated yield.

Additionally, we examined the reusability and recyclability of the catalyst (Figure 4). The aqueous layer containing the catalyst was decanted and reused for the next run under the same condition. As is shown in Figure 4, the yields of the product remained essentially constant for the 5 successive cycles higher than 95% yield, indicating superb stability and reusability of the catalyst.

### Versatility of the Substrates

Using the optimized reaction conditions (water, 70°C, 10 mol% catalyst), the generality and diversity of our methodology was evaluated by anthranilamide with a wide range of substituted aromatic aldehydes and the results are shown in Table 2. The reaction preceded smoothly using aldehydes bearing either electron-donating or electron-withdrawing groups to give the corresponding products. Aromatic aldehydes bearing strong electron-withdrawing groups (–NO_2_) could promote the reaction and offered better yields than those of aldehydes with electron-donating groups (OH, OMe) or halogen-substituted (-Br, -Cl). For aldehydes containing the electron-withdrawing group (acetylamino- and acetoxy-), the reaction rate was relatively fast and the yield of the product was also higher due to the electron deficiency. Heterocyclic aldehydes (-pyridine) offered the corresponding products in good yields. Nevertheless, *o*-trifluoromethyl substituted benzaldehyde only afforded 65% target product after 1 h, which may attribute to the disadvantage of steric hindrance. To further expand the scope of the green method, a variety of multiple substituent aldehydes like 2,4-dimethoxybenzaldehyde and 4-hydroxy-3,5-dimethoxybenzaldehyde were explored in this reaction under the optimized reaction conditions and found the formation of target products in good yield.

**Table 2:** Substrate scope of the reaction^a^.

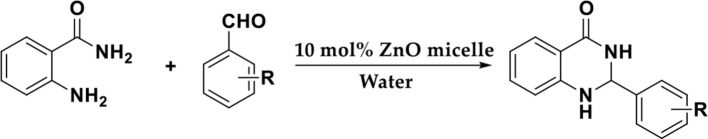			
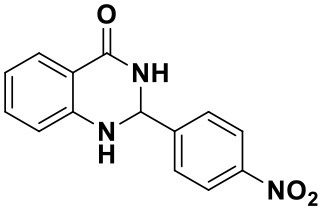	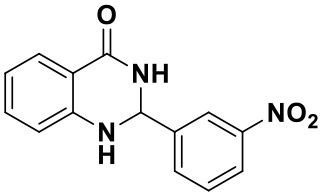	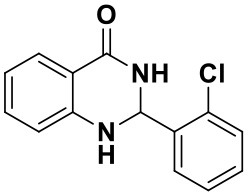	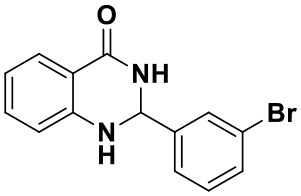
3a (10 min, 99%)	3b (8 min, 99%)	3c (20 min, 99%)	3d (22 min, 96%)
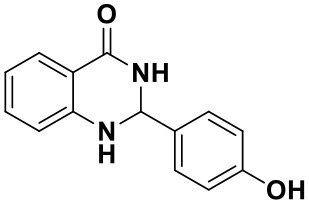	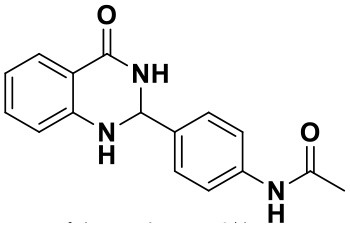	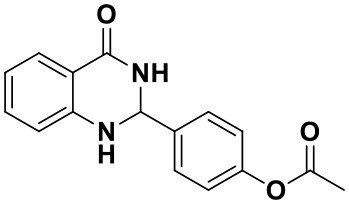	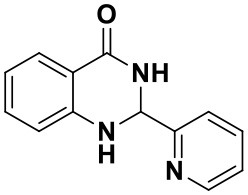
3e (50 min, 92%)	3f (40 min, 99%)	3g (50 min, 94%)	3h (30 min, 88%)
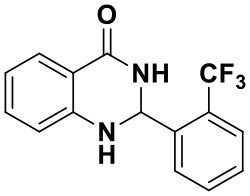	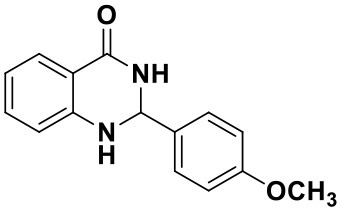	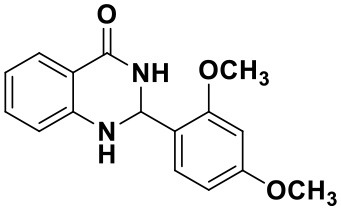	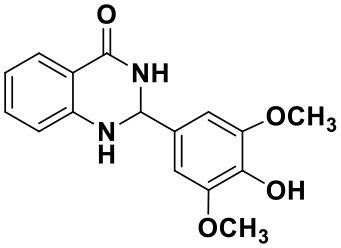
3i (30 min, 92%)	3j (52 min, 85%)	3k (60 min, 99%)	3i (50 min, 97%)

a *Reactions were carried out with benzaldehyde (1.2 mmol), anthranilamide (1 mmol) and reverse ZnO nanomicelles (10 mol%) in water (5 mL) at 70°C*.

### Advantages of Reverse Zinc Oxide Nanomicelles Catalyst System

Comparison of the reverse zinc oxide nanomicelles-catalyzed preparation of 2,3-dihydroquinazolin-4(1H)-ones in water with a range of other strategies demonstrated the high yields, low consumption of the catalyst, short reaction times, and the eco-friendly nature of the protocol (Table 3).

**Table 3:** Comparison of nanomillce catalyst with reported procedure.

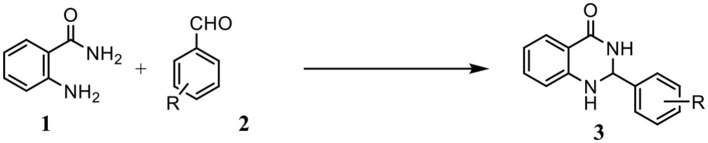							
**Entry**	**R**	**Catalyst (mol%)**	**Solvent**	**Temp. (°C)**	**Time (min)**	**Yield (%)**	**References**
1	4-NO_2_	Cu(I)-SBA-15	CH_2_Cl_2_	25	50	80	Hajjami et al., 2017
2	3-NO_2_	Pd(0)-SMT-MCM-41	EtOH	80	150	94	Noori et al., 2017
3	2-Cl	Br-TBA-Fe_3_O_4_	Water	70	60	91	Shiri et al., 2017
4	3-Br	Carbon dots	CH_3_CN	40	75	74	Majumdar et al., 2017
5	4-OH	Phosphatidylcholine nanoliposomes	Water	80	60	91	Tamaddon and Kazemivarnamkhasti, 2016
6	4-NHCOCH_3_	Indion Ina 225H	Water	20	180	90	Satish Reddy et al., 2015
7	4-OCOCH_3_	Basic ion liquid	Choline chloride	20	240	80	Obaiah et al., 2014
8	2-pyridin-2-yl	thiamine hydrochloride	Water	Reflux	25	80	Devi et al., 2017
9	2-CF_3_	/	CH_2_Cl_2_	Reflux	3 day	98	Zheng et al., 2013
10	4-OCH_3_	g-C_3_N_4_	EtOH	Reflux	15	91	Ghafuri et al., 2019
11	4-NO_2_	ChSO_3_HC	Water	r.t.	62	80	Azizi and Shirdel, 2017
12	3,5-OCH_3_-4-OH	p-sulfonic acid calix[4]arene	Water	20	24	84	Rahman et al., 2015
13	4-NO_2_	ZnO nanomicelles	Water	70	10	99	This work

### Plausible Mechanism

**Scheme 3:**
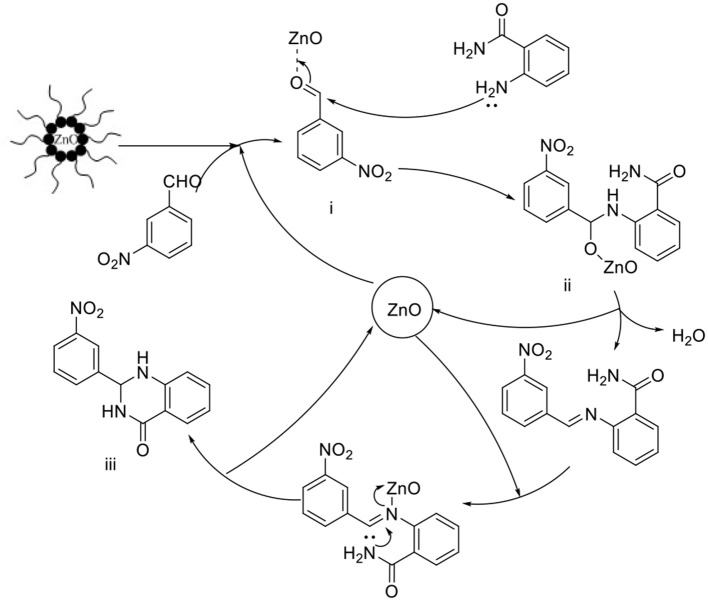
Plausible mechanism for ZnO nanomicelles catalyzed cyclocondensation reaction of **3a**.

The plausible mechanism for the formation of reverse zinc oxide nanomicelles-catalyzed synthesis of 2,3-dihydroquinazolin-4(1H)-ones in water is shown in Scheme 3. In this case, benzaldehyde prefers for the interior of the reverse micelles to coordinate with ZnO oxide, whereas the more polar anthranilamide reside on average in the head group region of the reverse micelles. Firstly, the coordination compound of zinc oxide-aldehyde intermediate (i) is formed. Subsequently, the condensation of the zinc oxide-aldehyde intermediate (i) with the anthranilamide produces imine intermediate (ii) after the dehydration and removal of zinc oxide. Finally, intermediate (i) was activated after ZnO connected to it and the following condensation of the imine with the amino group of anthranilamide produced the target product (iii) and released zinc oxide.

It is suspected that the inherent surface acidity of zinc oxide nanomicelles activates the benzaldehyde carbonyl carbon, making the carbon center highly electrophilic for the nucleophilic addition of anthranilamide. Hydrogen transfer resulted in protonated N, O-hemiketal followed by anchimeric assistance by the –NH_2_ group to give an imine which further undergoes intramolecular cyclization and deprotonation to give the desired dihydroquinazolinone product.

Reverse nanomicelles played an important role in combining the substrates, stabilizing the intermediate and promoting the dehydration condensation under neutral conditions in water catalyzed by the cationic water pool encapsulated in reverse micelles. The enhanced surface area due to nano particle size is an added advantage for its reactivity. Water promotes the nucleophilic addition reaction between imine intermediate and anthranilamide for the high polarity. All of these important factors are responsible for the high accessibility of the substrate molecules on the catalyst surface. Thus, the reaction occurred more easily in a micelles special with respect to its functioning as a nanoreactor.

## Conclusions

In conclusion, an efficient and convenient procedure for 2,3-dihydroquinazolin-4(1H)-ones has been developed via one-pot synthesis from anthranilamide and benzaldehyde. Reverse ZnO nano micelles were employed as catalyst and water was used as a green solvent for this transformation, producing excellent yields without the formation of by-products. The advantages of this method include atom economy, as well as being environmentally benign. The results prove the crucial role of reverse ZnO micelles as nanoreactors and will find more extensive applications in the field of green chemistry.

## Data Availability Statement

All datasets generated for this study are included in the article/supplementary material.

## Author Contributions

JM: conceptualization. JM and NC: methodology. YZ, HQ, and SM: formal analysis. NC and RX: data curation. JM and NC: writing-original draft preparation. JM: writing-review and editing. DP: supervision. DP and JM: funding acquisition.

## Conflict of Interest

The authors declare that the research was conducted in the absence of any commercial or financial relationships that could be construed as a potential conflict of interest.

## References

[B1] Azizi N. Shirdel F. (2017). Cholinesulfuric acid ionic liquid catalyzed an eco-friendly synthesis of 2,3-dihydroquinazolin-4(1H)-one in aqueous media. Bioorg. Med. Chem. 43, 3873–3882. 10.1007/s11164-016-2849-4

[B2] Bie Z. Li G. Wang L. Lv Y. Niu J. Gao S. (2016). A facile vanadium-catalyzed aerobic oxidative synthesis of quinazolinones from 2-aminobenzamides with aldehydes or alcohols. Tetrahedron Lett. 57, 4935–4938. 10.1016/j.tetlet.2016.09.077

[B3] Curran C. D. Lu L. Jia Y. Kiely C. J. Berger B. W. McIntosh S. (2017). Direct single-enzyme biomineralization of catalytically active ceria and ceria-zirconia nanocrystals. ACS Nano 11, 3337–3346. 10.1021/acsnano.7b00696 28212489

[B4] Devi J. Kalita S. J. Deka D. C. (2017). Expeditious synthesis of 2,3-dihydroquinazolin-4(1H)-ones in aqueous medium using thiamine hydrochloride (VB1) as a mild, efficient, and reusable organocatalyst. Synth. Commun. 47, 1601–1609. 10.1080/00397911.2017.1337149

[B5] Dindulkar S. D. Oh J. Arole V. M. Jeong Y. T. (2014). Supported ceric ammonium nitrate: a highly efficient catalytic system for the synthesis of diversified 2,3-substituted 2,3-dihydroquinazolin-4(1H)-ones. CR Chim. 17, 971–979. 10.1016/j.crci.2013.11.008

[B6] Dohle W. Jourdan F. L. Menchon G. Prota A. E. Foster P. A. Mannion P.. (2018). Quinazolinone-based anticancer agents: synthesis, antiproliferative SAR, antitubulin activity, and tubulin co-crystal structure. J. Med. Chem. 61, 1031–1044. 10.1021/acs.jmedchem.7b01474 29227648

[B7] Dutta A. Damarla K. Bordoloi A. Kumar A. Sarma D. (2019). KOH/DMSO: a basic suspension for transition metal-free tandem synthesis of 2,3-dihydroquinazolin-4(1H)-ones. Tetrahedron Lett. 60, 1614–1619. 10.1016/j.tetlet.2019.05.030

[B8] Ghafuri H. Goodarzi N. Rashidizadeh A. Douzandegi Fard M. A. (2019). ompg-C_3_N_4_/SO_3_H: an efficient and recyclable organocatalyst for the facile synthesis of 2,3-dihydroquinazolin-4(1H)-ones. Res. Chem. Intermediat. 45, 5027–5043. 10.1007/s11164-019-03873-6

[B9] Hajjami M. Ghorbani F. Yousofvand Z. (2017). Copper(I) complex of 1,3-dimethylbarbituric acid modified SBA-15 and its catalytic role for the synthesis of 2,3-dihydroquinazolin-4(1H)-ones and imidazoles. Appl. Organomet. Chem. 31:e3843 10.1002/aoc.3843

[B10] Hour M. J. Huang L. J. Kuo S. C. Xia Y. Bastow K. Nakanishi Y.. (2000). 6-alkylamino- and 2,3-dihydro-3'-methoxy-2-phenyl-4-quinazolinones and related compounds: their synthesis, cytotoxicity, and inhibition of tubulin polymerization. J. Med. Chem. 43, 4479–4487. 10.1021/jm000151c 11087572

[B11] Katla R. Chowrasia R. Da Silva C. De Oliveira A. Dos Santos B. Domingues N. (2017). Recyclable [Ce(l-Pro)2]2 (oxa) used as heterogeneous catalyst: one-pot synthesis of 2,3-dihydroquinazolin-4(1H)-ones in ethanol. Synthesis 49, 5143–5148. 10.1055/s-0036-1590886

[B12] Katoh T. Takai T. Yukawa T. Tsukamoto T. Watanabe E. Mototani H.. (2016). Discovery and optimization of 1,7-disubstituted-2,2-dimethyl-2,3-dihydroquinazolin-4(1H)-ones as potent and selective PKCθ inhibitors. Bioorg. Med. Chem. 24, 2466–2475. 10.1016/j.bmc.2016.04.008 27117263

[B13] Khan I. Zaib S. Batool S. Abbas N. Ashraf Z. Iqbal J.. (2016). Quinazolines and quinazolinones as ubiquitous structural fragments in medicinal chemistry: an update on the development of synthetic methods and pharmacological diversification. Bioorg. Med. Chem. 24, 2361–2381. 10.1016/j.bmc.2016.03.031 27112448

[B14] Khoshnavazi R. Bahrami L. Havasi F. (2016). Organic–inorganic hybrid polyoxometalate and its graphene oxide–Fe_3_O_4_ nanocomposite, synthesis, characterization and their applications as nanocatalysts for the knoevenagel condensation and the synthesis of 2,3-dihydroquinazolin-4(1H)-ones. RSC Adv. 6, 100962–100975. 10.1039/C6RA15339A

[B15] Kshirsagar U. A. (2015). Recent developments in the chemistry of quinazolinone alkaloids. Org. Biomol. Chem. 13, 9336–9352. 10.1039/C5OB01379H 26278395

[B16] Liu Z. Zeng L-Y. Li C. Yang F. Qiu F. Liu S.. (2018). “On-water” synthesis of quinazolinones and dihydroquinazolinones starting from o-bromobenzonitrile. Molecules 23:2325. 10.3390/molecules23092325 30213061 PMC6225144

[B17] Majumdar B. Mandani S. Bhattacharya T. Sarma D. Sarma T. K. (2017). Probing carbocatalytic activity of carbon nanodots for the synthesis of biologically active dihydro/spiro/glyco quinazolinones and aza-michael adducts. J. Org. Chem. 82, 2097–2106. 10.1021/acs.joc.6b02914 28121145

[B18] Mou J. Gao G. Chen C. Liu J. Gao J. Liua Y. (2017). Highly efficient one-pot three-component betti reaction in water using reverse zinc oxide micelles as a recoverable and reusable catalyst. RSC Adv. 7, 13868–13875. 10.1039/C6RA28599F

[B19] Noori N. Nikoorazm M. Ghorbani-Choghamarani A. (2017). Synthesis and characterization of Pd(0)-SMT-MCM-41 and its application in the amination of aryl halides and synthesis of 2,3-dihydroquinazolin-4(1H)-ones as efficient and recyclable nanostructural catalyst. Catal. Lett. 147, 204–214. 10.1007/s10562-016-1905-4

[B20] Obaiah O. Nandeesh K. Raghavendra G. M. Siddalingaiah Chottanahalli P. Subbegowda Kanchugarakoppal R. (2014). Synthesis of 2-aryl substituted 2,3-dihydroquinazoline-4(1H)-ones under solvent free conditions using ionic liquid as a mild and efficient catalyst. Eur. J. Chem. 5, 671–675. 10.5155/eurjchem.5.4.671-675.1071

[B21] Parua S. Das S. Sikari R. Sinha S. Paul N. D. (2017). One-pot cascade synthesis of quinazolin-4(3H)-ones via nickel-catalyzed dehydrogenative coupling of o-aminobenzamides with alcohols. J. Org. Chem. 82, 7165–7175. 10.1021/acs.joc.7b00643 28653839

[B22] Pathare R. S. Maurya A. K. Kumari A. Agnihotri V. K. Verma V. P. Sawant D. M. (2019). Synthesis of quinazoline-3-oxides via a Pd(ii) catalyzed azide-isocyanide coupling/cyclocondensation reaction. Org. Biomol. Chem. 17, 363–368. 10.1039/C8OB02627K 30556560

[B23] Patil A. Barge M. Rashinkar G. Salunkhe R. (2015). Aqueous hydrotrope: an efficient and reusable medium for a green one-pot, diversity-oriented synthesis of quinazolinone derivatives. Mol. Divers. 19, 435–445. 10.1007/s11030-015-9580-8 25790788

[B24] Pospisilova J. Krchnak V. Schutznerova E. (2018). Traceless solid-phase synthesis of 1'H-spiro[pyrrolidine-3,2'-quinazolin]-2-ones and 1'H-spiro[piperidine-3,2'-quinazolin]-2-ones via lactamization of 1,2-dihydroquinazoline-2-carboxylates. ACS Comb. Sci. 21, 1–5. 10.1021/acscombsci.8b00145 30485058

[B25] Punnoose A. Dodge K. Rasmussen J. W. Chess J. Wingett D. Anders C. (2014). Cytotoxicity of ZnO nanoparticles can be tailored by modifying their surface structure: a green chemistry approach for safer nanomaterials. ACS Sustain. Chem. Eng. 2, 1666–1673. 10.1021/sc500140x 25068096 PMC4105193

[B26] Rahman M. Ling I. Abdullah N. Hashim R. Hajra A. (2015). Organocatalysis by p-sulfonic acid calix[4]arene: a convenient and efficient route to 2,3-dihydroquinazolin-4(1H)-ones in water. RSC Adv. 5, 7755–7760. 10.1039/C4RA16374E

[B27] Ramesh R. Nagasundaram N. Meignanasundar D. Vadivel P. Lalitha A. (2017). Glycerol assisted eco-friendly strategy for the facile synthesis of 4,4′-(arylmethylene)bis(3-methyl-1H-pyrazol-5-ols) and 2-aryl-2,3-dihydroquinazolin-4(1H)-ones under catalyst-free conditions. Res.Chem. Intermediat. 43, 1767–1782. 10.1007/s11164-016-2728-z

[B28] Rostamizadeh S. Amani A. M. Mahdavinia G. H. Amiri G. Sepehrian H. (2010). Ultrasound promoted rapid and green synthesis of 1,8-dioxo-octahydroxanthenes derivatives using nanosized MCM-41-SO_3_H as a nanoreactor, nanocatalyst in aqueous media. Ultrason. Sonochem. 17, 306–309. 10.1016/j.ultsonch.2009.10.004 19913449

[B29] Rostamnia S. Xin H. (2014). Basic isoreticular metal-organic framework (IRMOF-3) porous nanomaterial as a suitable and green catalyst for selective unsymmetrical hantzsch coupling reaction. Appl. Organomet. Chem. 28, 359–363. 10.1002/aoc.3136

[B30] Saeedi M. Mohammadi-Khanaposhtani M. Pourrabia P. Razzaghi N. Ghadimi R. Imanparast S.. (2018). Design and synthesis of novel quinazolinone-1,2,3-triazole hybrids as new anti-diabetic agents: *in vitro* α-glucosidase inhibition, kinetic, and docking study. Bioorg. Chem. 83, 161–169. 10.1016/j.bioorg.2018.10.023 30366316

[B31] Sanles-Sobrido M. Perez-Lorenzo M. Rodriguez-Gonzalez B. Salgueirino V. Correa-Duarte M. A. (2012). Highly active nanoreactors: nanomaterial encapsulation based on confined catalysis. Angew. Chem. Int. Ed. Engl. 51, 3877–3882. 10.1002/anie.201105283 22307952

[B32] Sarfraz M. Sultana N. Rashid U. Akram M. S. Sadiq A. Tariq MI. (2017). Synthesis, biological evaluation and docking studies of 2,3-dihydroquinazolin-4(1H)-one derivatives as inhibitors of cholinesterases. Bioorg. Chem. 70, 237–244. 10.1016/j.bioorg.2017.01.004 28126287

[B33] Satish Reddy K. Tigulla P. Santosh Kumar K. Satyender A. (2015). Green protocol for the synthesis of 2-aryl-2,3-dihydroquinazoline-4(1H)-ones using indion ina 225H resin. Asian J. Chem. 27, 2222–2224. 10.14233/ajchem.2015.18362

[B34] Shaabani A. Maleki A. Mofakham H. (2008). Click reaction: highly efficient synthesis of 2,3-dihydroquinazolin-4(1H)-ones. Synthetic Commun. 38, 3751–3759. 10.1080/00397910802213802

[B35] Shiri L. Ghorbani-Choghamarani A. Kazemi M. (2017). Synthesis and characterization of bromine source supported on magnetic Fe_3_O_4_ nanoparticles: a new, versatile and efficient magnetically separable catalyst for organic synthesis. Appl. Organomet. Chem. 31:e3634 10.1002/aoc.3634

[B36] Singh M. Raghav N. (2015). 2,3-Dihydroquinazolin-4(1H)-one derivatives as potential non-peptidyl inhibitors of cathepsins B and H. Bioorg. Chem. 59, 12–22. 10.1016/j.bioorg.2015.01.005 25665518

[B37] Sivaguru P. Parameswaran K. Lalitha A. (2017). Antioxidant, anticancer and electrochemical redox properties of new bis(2,3-dihydroquinazolin-4(1H)-one) derivatives. Mol. Divers. 21, 611–620. 10.1007/s11030-017-9748-5 28477101

[B38] Sultana N. Sarfraz M. Tanoli S. T. Akram M. S. Sadiq A. Rashid U. (2017). Synthesis, crystal structure determination, biological screening and docking studies of N(1)-substituted derivatives of 2,3-dihydroquinazolin-4(1H)-one as inhibitors of cholinesterases. Bioorg. Chem. 72, 256–267. 10.1016/j.bioorg.2017.04.009 28495556

[B39] Sun J. W. Tao T. Xu D. Cao H. Kong Q. G. Wang X. (2018). Metal-free oxidative cyclization of 2-amino-benzamides, 2-aminobenzenesulfonamide or 2-(aminomethyl)anilines with primary alcohols for the synthesis of quinazolinones and their analogues. Tetrahedron Lett. 59, 2099–2102. 10.1016/j.tetlet.2018.04.054

[B40] Tamaddon F. Kazemivarnamkhasti M. (2016). Self-assembled nanoliposomes of phosphatidylcholine: bridging the gap between organic and aqueous media for a green synthesis of hydroquinazolinones. Synletter 27, 2510–2514. 10.1055/s-0035-1562604

[B41] Wang Y. Wu H. Wu W-N. Li S.-J. Xu Z-H. Xu Z-Q. (2018). An AIRE active schiff base bearing coumarin and pyrrole unit: Cu^2+^ detection in either solution or aggregation states. Sensors Actuat. B Chem. 260, 106–115. 10.1016/j.snb.2017.12.201

[B42] Wu Y. Wang F. Zhan S. Liu L. Luo Y. Zhou P. (2013). Regulation of hemin peroxidase catalytic activity by arsenic-binding aptamers for the colorimetric detection of arsenic(iii). RSC Adv. 3:25614 10.1039/c3ra44346a

[B43] Xing J. Yang L. Yang Y. Zhao L. Wei Q. Zhang J.. (2017). Design, synthesis and biological evaluation of novel 2,3-dihydroquinazolin- 4(1H)-one derivatives as potential fXa inhibitors. Eur. J. Med. Chem. 125, 411–422. 10.1016/j.ejmech.2016.09.055 27689724

[B44] Zawawi N. K. Rajput S. A. Taha M. Ahmat N. Ismail N. H. Abdullah N.. (2015). Benzimidazole derivatives protect against cytokine-induced apoptosis in pancreatic β-cells. Bioorg. Med. Chem. Lett. 25, 4672–4676. 10.1016/j.bmcl.2015.08.022 26330080

[B45] Zhang H. Liu H. Luo X. Wang Y. Liu Y. Jin H.. (2018). Design, synthesis and biological activities of 2,3-dihydroquinazolin-4(1H)-one derivatives as TRPM2 inhibitors. Eur. J. Med. Chem. 152, 235–252. 10.1016/j.ejmech.2018.04.045 29723786

[B46] Zheng Y. Bian M. Deng X-Q. Wang S-B. Quan Z-S. (2013). Synthesis and anticonvulsant activity evaluation of 5-phenyl-[1,2,4]triazolo[4,3-c]quinazolin-3-amines. Arch. Pharm. Med. Chem. 346, 119–126. 10.1002/ardp.201200376 23255333

